# Lignan formation in hairy root cultures of Edelweiss (*Leontopodium nivale* ssp. *alpinum* (Cass.) Greuter)

**DOI:** 10.1016/j.fitote.2014.06.008

**Published:** 2014-09

**Authors:** Christoph Wawrosch, Stefan Schwaiger, Hermann Stuppner, Brigitte Kopp

**Affiliations:** a Department of Pharmacognosy, University of Vienna, Althanstr. 14, A-1090 Vienna, Austria; b Institute of Pharmacy/Pharmacognosy, University of Innsbruck, Josef-Moeller-Haus, Innrain 52c, A-6020 Innsbruck, Austria

**Keywords:** LG, leoligin, MLG, 5-methoxy-leoligin, MS, nutrient medium after Murashige and Skoog (1962), MeJa, methyl jasmonate, YE, yeast extract, *Leontopodium nivale* ssp. *alpinum*, Edelweiss, Leoligin, 5-Methoxy-leoligin, Lignan, *Agrobacterium rhizogenes*, Hairy roots, Elicitor

## Abstract

A hairy root line of Edelweiss (*Leontopodium nivale* ssp. *alpinum* (Cass.) Greuter) was obtained upon transformation with *Agrobacterium rhizogenes* strain ATCC15834. Elicitation of this line with silver nitrate, sucrose, methyl jasmonate and yeast extract at various concentrations in most cases resulted in a stimulation of lignan biosynthesis. Through elicitation with 6% sucrose the roots accumulated the pharmacologically active lignans leoligin and 5-methoxy-leoligin at levels of 0.0678% and 0.0372%, respectively, without significant growth inhibition. These lignan levels were comparable to those found in intact roots of cultivated Edelweiss. The biotechnological production of leoligin could be an attractive option for the continuous, field culture-independent production of the valuable secondary metabolites leoligin and 5-methoxy-leoligin.

## Graphical abstract

**Figure:**
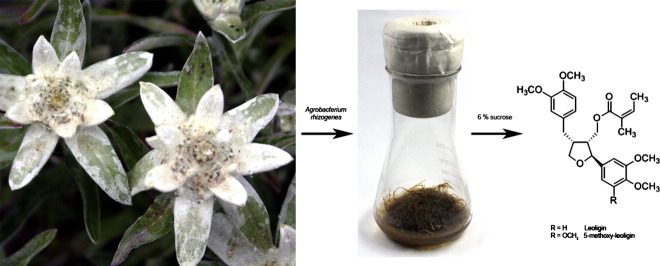

## 1 Introduction

**Fig. 1:**
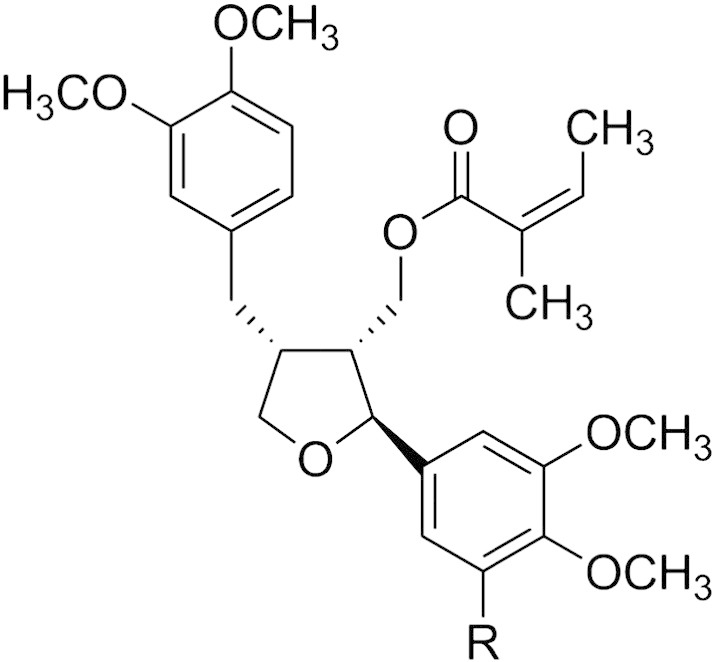
Structure of leoligin (R = H) and 5-methoxy-leoligin (R = OCH_3_).

The genus *Leontopodium* (R.Br. ex Cassini) belongs to the Asteraceae family and comprises about 30 species [1]. The well-known alpine Edelweiss (*Leontopodium nivale* ssp. *alpinum* (Cass.) Greuter or *L. alpinum* Cass.) is found in the mountain regions of Europe, mostly in the Alps, Carpathians, Pyrenees, the Tatra and the Balkan Peninsula [2]. Edelweiss has been used in traditional medicine since centuries against ailments like diarrhea, abdominal aches, bronchitis or fever [3], [4]. Recent studies revealed that extracts from aerial parts and roots possess anti-inflammatory [5], [6] and analgesic [6] activities. Phytochemical research has revealed the occurrence of various secondary compounds like diterpenes [7], sesquiterpenes [8], [9], benzofuranoids [10] and lignans [7], [10]. In the latter class the metabolite, leoligin, has recently been isolated [10]. This lariciresinol-type lignan (see Fig. 1) has been shown to inhibit in vitro leukotriene biosynthesis and intimal hyperplasia of venous bypass grafts, seemingly without toxic side effects [11]. It inhibits in vivo neointima formation without causing endothelial damage, and it is not thrombogenic [11]. Vein graft disease, i.e. the progressive degeneration of veins used in surgical bypass operations, is characterized by endothelial damage, smooth muscle cell proliferation and pro-inflammatory signaling [11], [12]. Drug eluting stents are considered to be the most likely approach in the prevention of graft failure, but currently there is a lack of drugs with specific activity. Leoligin is considered to have a great potential for the treatment of vein graft disease [11]. The structurally related compound 5-methoxy-leoligin (see Fig. 1) has recently been shown to be a very promising candidate for the development of the first low molecular weight pro-angiogenic and pro-arteriogenic drug for the treatment of myocardial infarction [13]. Myocardial infarction is a major cause of mortality worldwide. Recent treatment strategies in the therapy of myocardial infarction aim at the improvement of ventricular function, among others by stimulating angiogenesis and arteriogenesis, namely the induction of artery growth to bypass occluded arteries [13]. In this, there is an urgent need to find new drugs, and 5-methoxy-leoligin has shown to possess corresponding pro-angiogenic and pro-arteriogenic activities.

Edelweiss is a protected plant in many countries. While it is cultivated in large quantities in Switzerland [14], the isolation of relevant amounts from Edelweiss roots remains a laborious task due to the low content [10], [15] and the thin and fibrous nature of the roots of cultivated plants [16]. As the chemical synthesis has not yet been described the biotechnological production might be an alternative approach to the procurement of relevant amounts of these lignans. Hairy root cultures, i.e. in vitro cultured roots which result from the infection (transformation) of higher plants with the soil-born bacterium *Agrobacterium rhizogenes*, have been investigated for a few decades as biological systems for the production of secondary compounds from medicinal plants [17]. Hairy roots can in many cases produce the same compounds found in normal roots of the parent plant, but while in callus or cell suspension cultures this productivity frequently diminishes over time it remains stable in transformed roots [18]. Furthermore, in the recent past hairy root technology has been significantly improved concerning accumulation and excretion of secondary metabolites after elicitation, and scale-up of the culture process [17]. With regard to the production of lignans from hairy roots a number of studies have focused on podophyllotoxin and its derivatives, an important lead for anticancer drugs. For example, hairy root lines of *Linum album* produced 105 μg/L [19] or 5.12 mg/L [20] of the lignan while a content of 14.11 mg/L was measured in the roots of wild growing plants [19]. In *Linum flavum*, hairy roots were reported to contain up to 3.5% 5-methoxypodophyllotoxin [21] which is comparable to the amount of 3.68% described for roots of greenhouse grown plants [22]. Another arylnaphthalene lignan, justicin B, is found in normal root cultures (12.5 mg/L) and hairy root cultures (16.9 mg/L) of *Linum austriacum*[23]. Silymarin is a flavonolignan complex with hepatoprotective properties isolated from the fruits of the milk thistle plant, *Silybum marianum*. While hairy roots contained more isosilybin A and B than untransformed root cultures, the content of four other flavonolignans was higher in the latter culture type [24].

It has been demonstrated previously that *L. nivale* ssp. *alpinum* can be transformed with *A. rhizogenes*, and resulting hairy root lines were shown to produce anthocyanins, hydroxycinnamic acid esters, and essential oil [25]. In the present study a hairy root clone of Edelweiss was investigated specifically in respect of its leoligin and 5-methoxy-leoligin content and the influence of the treatment with elicitors, molecules that stimulate defense or stress-induced responses in plants [26], on product formation.

## 2 Experimental

### 2.1 Plant material

Seeds of *L. nivale* ssp. *alpinum* were purchased from Austrosaat AG (Vienna, Austria) and were surface sterilized for 30 min with an aqueous NaOCl solution (3.4% active chlorine). They were then aseptically germinated on modified semisolid half-strength Murashige and Skoog (MS) medium [27] supplemented with 1% sucrose and 0.3% Gelrite® (Carl Roth, Karlsruhe, Germany). Shoots of 4 weeks old seedlings were subsequently transferred to modified semisolid MS medium with 3% sucrose, 0.4 mg/L thiamine hydrochloride, 80 mg/L myo-inositol, 100 mg/L caseine hydrolysate, 0.7% agar (Merck, Darmstadt, Germany), 0.55 μM 1-naphthaleneacetic acid and 0.25 μM kinetin [28] for further multiplication. Every 4 weeks shoot clusters were divided into single shoots which were transferred to fresh medium. All cultures were kept at 25 ± 1 °C under a 16 hour photoperiod with a light intensity of 40 μM · m^− 2^ · s^− 1^ (Sylvania Gro-Lux® fluorescent tubes). A number of seedlings of this seed batch were also potted into gardening soil and grown to the flowering stage. Voucher specimens and living plants are kept at the Department of Pharmacognosy, University of Vienna.

Three normal root samples of cultivated *L. nivale* ssp. *alpinum* plants were obtained from the Mediplant Swiss Research Centre on Medicinal and Aromatic Plants, Conthey, Switzerland (two samples) and from W. Faulhammer, Innsbruck, Austria (one sample). Vouchers are stored at the herbarium of the Institute of Pharmacy/Pharmacognosy, University of Innsbruck.

### 2.2 Hairy root culture

*A. rhizogenes* strain ATCC15834 was grown in liquid YMB medium [29]. The middle veins of leaves of in vitro grown Edelweiss shoots were gently scratched with a scalpel dipped in the bacterial suspension and the shoots where then cultivated on modified semisolid half-strength MS medium with 3% sucrose in the light. Single roots which emerged from the wounded sites after 4 weeks in average were dissected from the leaves and treated twice for 10 days each with liquid modified MS medium containing 3% sucrose and 500 mg/L of the antibiotic, cefotaxim. Subsequently the hairy roots were routinely transferred to liquid modified MS medium with 3% sucrose every 4 weeks: An inoculum of ca. 0.5 g was transferred to 50 mL of medium in a 250 mL Erlenmeyer flask and cultivated at 25 ± 1 °C in the dark on a rotary shaker at 100 RPM. The root line K8A was used for the elicitation experiments.

### 2.3 Elicitor treatments

Elicitors were added to 3 weeks old hairy root cultures except for the treatments with elevated sucrose concentrations which occurred for the whole culture period. Silver nitrate (Merck, Darmstadt, Germany, analytical grade) was dissolved in distilled water and the filter sterilized (0.22 μm) solution was added to hairy root cultures at final concentrations of 15, 30 and 60 μM AgNO_3_, respectively. Similarly, filter sterilized solutions of methyl jasmonate (MeJa; Sigma–Aldrich, St. Louis, USA; 95% purity) in 96% ethanol were added to achieve final concentrations of 50, 100, 200 and 300 μM. Yeast extract (YE; Sigma–Aldrich, St. Louis, USA) was purified by dual precipitation with ethanol as described by Hahn and Albersheim [30]. After autoclaving (20 min at 121 °C) of the resulting aqueous solution aliquots were added to hairy root cultures at final concentrations of 1, 2 or 5 g/L. For osmotic treatment, hairy roots were inoculated in liquid modified MS media supplemented with 5, 6 or 7% sucrose. Root materials were always harvested after a total cultivation period of 4 weeks and were dried at room temperature.

### 2.4 HPLC-UV analysis

#### 2.4.1 Sample preparation

A small amount of ground root sample was weighted (100.0 mg). The plant material was sonicated with 20 mL of dichloromethane for 10 min in the ultrasonic bath before being filtrated over cotton wool. This manipulation was repeated two times with 10 mL of dichloromethane each. After evaporation of the solvent of the combined extracts at reduced pressure the dry residue was dissolved in 1.00 mL of methanol, again filtered over cotton wool and analyzed by HPLC-UV. The efficacy of the extraction protocol was proved by a fourth extraction with 10 mL dichloromethane. The extract was evaporated separately, dissolved in 1.00 mL MeOH and analyzed by HPLC-UV. Since no signals of leoligin and 5-methoxy-leoligin were found, extraction was found to be exhaustive. Two extracts of each hairy root culture sample were prepared. Both were analyzed three times, and the six values of the peak area were used to perform the quantification of leoligin and 5-methoxy-leoligin in the samples.

#### 2.4.2 HPLC-UV method and quantification parameters

An HP 1050 system (Agilent, Waldbronn, Germany) equipped with auto sampler, DAD and column thermostat was used. The stationary phase was a Phenomenex Kinetex 2.6 μ C18 100 A (100 mm × 2.1 mm) with 2.6 μm particles equipped with the corresponding guard column. The temperature was set to 40 °C. The mobile phase consisted of two solvents: A = water, B = acetonitrile. The composition during run was set as following: 0 min: 65% A, 35% B; 20 min: 50% A, 50% B; 25 min: 1% A, 99% B; stop: 35 min; post time: 15 min. The flow rate was 0.250 mL/min and the detection wavelength was 205 nm. As standard for quantification pure leoligin and 5-methoxy-leoligin were dissolved in 1.00 mL of methanol and diluted to obtain five reference solutions (73.1 μg/mL to 4.6 μg/mL; 54.4 μg/mL to 3.4 μg/mL), which were analyzed to afford a calibration curve of y = 230818.83405x + 496.66528; R^2^ = 0.99819 for leoligin as well as y = 190969.00754x − 47.98333; R^2^ = 0.9995219 for 5-methoxy-leoligin.

#### 2.4.3 Chemicals and reagents

All solvents (dichloromethane, methanol and acetonitrile) were of analytical grade (99.9%) provided from Merck (Darmstadt, Germany). The used water was produced via reverse-osmosis. Leoligin and 5-methoxy-leoligin had a purity level higher than 98%, according to LC-DAD/MS and NMR examination. These references were prepared at the Institute of Pharmacy/Pharmacognosy of the University of Innsbruck [7].

## 3 Results and discussion

The hairy roots line K8A which was investigated in this study was chosen due to its fast growth. When cultivated under standard conditions, i.e. in liquid modified MS medium with 3% sucrose, it yielded 0.0062% LG and 0.0049 MLG (Table 1). In many cases, secondary metabolite accumulation in hairy root cultures can be enhanced by elicitation, i.e. treatment of the culture with biotic and abiotic elicitors [31]. We therefore treated our hairy root line with the two abiotic elicitors silver nitrate and sucrose, and the two biotic elicitors yeast extract and methyl jasmonate. As elicitation can, despite an improved product yield, also result in decrease of biomass accumulation [32], hairy root growth was assessed in terms of final dry weight, too. Silver nitrate at a concentration of 15 μM led to a ca. 5-fold increase of the levels of both LG and MLG, but on the other hand root growth was negatively influenced (about 30% less biomass). Elicitation with 30 or 60 μM AgNO_3_ had a less pronounced and not significant impact on lignan biosynthesis. Similarly, enhanced production of silymarin in hairy root cultures of *S. marianum* has also been achieved through the use of silver nitrate [33]. A treatment with 30 or 60 μM of this elicitor lowered the total phenolics content in hairy roots of *Salvia miltiorrhiza*, but slightly stimulated the formation of these metabolites at a concentration of 15 μM [31]. But, in the same culture type Ge and Wu [34] found Ag^+^ to stimulate the production of tanshinones, and silver nitrate significantly increased the accumulation of the tropane alkaloids, scopolamine and hyoscyamine in hairy roots of *Brugmansia candida*[35].

**Table 1:** Final biomass (g dry wt.) and contents (% dry wt.) of leoligin and 5-methoxy-leoligin in hairy root clone K8A of *Leontopodium nivale* ssp. *alpinum* treated with various elicitors.

Sample/treatment	Final biomass⁎ (g dry wt.)	Leoligin (w%)⁎⁎	5-methoxy-leoligin (w%)⁎⁎
K8A control	0.62 ± 0.03^ade^	0.0062 ± 0.0021^a^	0.0049 ± 0.0014^ab^
15 μM AgNO_3_	0.44 ± 0.03^bc^	0.0321 ± 0.0094^bc^	0.0260 ± 0.0075^e^
30 μM AgNO_3_	0.43 ± 0.02^bc^	0.0183 ± 0.0021^ab^	0.0153 ± 0.0019^bcd^
60 μM AgNO_3_	0.36 ± 0.03^b^	0.0217 ± 0.0015^ab^	0.0177 ± 0.0014^cde^
5% Sucrose	0.64 ± 0.04^ae^	0.0142 ± 0.0032^ab^	0.0083 ± 0.0018^abc^
6% Sucrose	0.59 ± 0.03^ade^	0.0678 ± 0.0042^d^	0.0372 ± 0.0025^f^
7% Sucrose	0.70 ± 0.05^a^	0.0221 ± 0.0062^ab^	0.0101 ± 0.0027^abcd^
1 g/L YE	0.69 ± 0.04^a^	0.0192 ± 0.0054^ab^	0.0118 ± 0.0028^abcd^
2 g/L YE	0.61 ± 0.05^ade^	0.0337 ± 0.0094^bc^	0.0204 ± 0.0050^de^
5 g/L YE	0.59 ± 0.05^ade^	0.0035 ± 0.0016^a^	0.0019 ± 0.0008^a^
50 μM MeJa	0.50 ± 0.05^cd^	0.0316 ± 0.0097^bc^	0.0116 ± 0.0028^abcd^
100 μM MeJa	0.45 ± 0.05^bc^	0.0498 ± 0.0152^cd^	0.0188 ± 0.0050^cde^
200 μM MeJa	0.54 ± 0.04^cde^	0.0249 ± 0.0037^ab^	0.0098 ± 0.0013^abcd^
300 μM MeJa	0.59 ± 0.03^ade^	0.0173 ± 0.0035^ab^	0.0113 ± 0.0021^abcd^

⁎ Values are mean ± S.E. (n = 5) per culture flask with 50 mL nutrient medium after 4 weeks of culture; means followed by the same letter are not significantly different (p = 0.05) according to Duncan's multiple range test.

⁎⁎ Values are mean ± S.E. (n = 2, each measured 3 times); means followed by the same letter are not significantly different (p = 0.05) according to Duncan's multiple range test.

Beside its role as carbon source in in vitro cultures, sucrose at elevated concentrations can act as abiotic elicitor due to increased osmotic pressure. We therefore investigated the effect of this sugar at concentrations of 5, 6 or 7%. While lignan formation was not significantly enhanced when 5 or 7% sucrose was used, at 6% the content of LG and MLG increased to 0.0678% and 0.0372%, respectively, which was 10.9-fold and 7.6-fold higher than that in the control roots with the standard sucrose concentration of 3%. Hairy root growth was not significantly impaired at any of the tested sucrose concentrations. An elevated sucrose concentration of 6% also stimulated glycyrrhizin production in hairy roots of *Glycyrrhiza inflata*[36]. In hairy roots of *Withania somnifera*, accumulation of the steroidal lactone, withaferin A was enhanced by treatment with 4% but not with 6% sucrose [37]. Plant cell suspension cultures of *Melastoma malabathricum* produced more anthocyanins with 4.5–7.5% sucrose than with 3% [38], and using 5% sucrose the formation of rosmarinic acid in suspension cultures of *Ocimum sanctum* was higher than with 4 or 7% of the sugar [39].

Yeast extract (YE) and preparations thereof have been widely used as elicitors in plant tissue culture [40]. When purified YE [30] was added to cultures of the Edelweiss hairy root clone K8A, 1 or 5 g/L did not significantly influence neither lignan formation nor root growth. At a concentration of 2 g/L a 5-fold (LG) and 4-fold (MLG) promotion of product formation was observed, while biomass increase was not affected. In cell cultures of *S. miltiorrhiza* 100 mg/L but not 50 or 200 mg/L YE led to the highest tanshinone yield [41]. In hairy root cultures of the same plant species rosmarinic acid formation was enhanced similarly irrespective of the concentration of YE elicitor, while cryptotanshinone biosynthesis was improved in a dose dependent manner [42].

As a second biotic elicitor we chose methyl jasmonate (MeJa) which has been shown to stimulate secondary metabolite formation in a wide variety of plant in vitro cultures. In particular, the production of lignans was shown to rise in cell suspension cultures of *Forsythia* x *intermedia*[43] and *L. album*[44] upon treatment with MeJa. Elicitation of Edelweiss hairy root clone K8A with 50 or 100 μM MeJa resulted in accumulation of up to 0.0498% LG and 0.0188% MLG (8-fold and 3.8-fold higher than in the control, respectively), although at 100 μM MeJa root growth was significantly suppressed. Higher concentrations (200 or 300 μM MeJa) showed no influence on biomass production but enhanced lignan formation only to a moderate non significant extent.

The leoligin content of wild growing Edelweiss has been described to account for 0.005–0.010% [15]. Within the present study, analysis of two samples of cultivated *L. nivale* ssp. *alpinum* from Switzerland (Mediplant Swiss Research Centre on Medicinal and Aromatic Plants) revealed a content of 0.0155 and 0.0547% LG and 0.1293 and 0.0672% MLG, respectively. Another sample from field culture in Innsbruck, Austria yielded 0.0632% LG and 0.0421% MLG. Although no further data concerning the lignan content in Edelweiss are available so far, a variability regarding both the content and the ration of LG to MLG seem to be likely. Furthermore, it is not clear whether the growing conditions (wild vs. controlled cultivation) have an influence on lignan production. Anyhow, the basic LG content of the hairy root clone K8A used in the present study was found to be of the order of wild growing Edelweiss [15]. Similarly, untransformed roots and hairy roots of *L. flavum* accumulated comparable amounts of the lignan, 5-methoxypodophyllotoxin [21], [22]. As delineated above, lignan contents in hairy roots have been described to be higher, lower or equal to that in normal roots depending on factors like e.g. plant genotype or *Agrobacterium* strain used for transformation. The production of LG could be significantly increased nearly 11-fold through elicitation with 6% sucrose. Similarly, the MLG content of clone K8A could be enhanced 7.6-fold, and elicitation had no significant impact on biomass accumulation.

## 4 Conclusions

Only recently the highly active lignan compounds leoligin and 5-methoxy-leoligin have been isolated from the roots of the alpine Edelweiss, casting new light on this tradition-steeped plant. As an alternative to the laborious extraction of the low amounts found in the plant, we could show that a transformed hairy root line of Edelweiss accumulates the valuable lignans in concentrations which, upon elicitation with 6% sucrose, resembles the content in normal roots. Analyses of three samples of normal roots revealed a degree of variation in both total lignan content and ratio of the two compounds. Future work will therefore focus on the establishment of further hairy root clones starting from high yielding Edelweiss genotypes, and using additional *A. rhizogenes* strains. In any case, as a continuous, sustainable and renewable production system independent of climatic or environmental effects [45] the biotechnological production of leoligin and 5-methoxy-leoligin would be of advantage over the extraction from field grown plants.
